# Supplementary material for: Synthesis, Structure, and Characterizations of a Volatile/Soluble Heterometallic Hexanuclear Precursor [NaMn2(thd)4(OAc)]2
Source: Molecules. 2023 Nov 27;28(23):7795. doi: 10.3390/molecules28237795 (PMC10708067; doi:10.3390/molecules28237795)
Supplement: Supplementary file 1 [file molecules-28-07795-s001.zip › molecules-2715349-supplementary.pdf]

Supplementary Information

Synthesis, Structure, and Characterizations of a Volatile/Soluble Heterometallic Hexanuclear Precursor $[NaMn_2(thd)_4(OAc)]_2$

Yuxuan Zhang , Zheng Wei , Evgeny V. Dikarev *

Table of Contents

1. Solid-State Structure of [$NaMn_2(thd)_4(OAc)]_2$ 3
2. IR Spectrum of [$NaMn_2(thd)_4(OAc)]_2$ 7
3. ^{1}H NMR spectrum of [Na(thd)] 8
4. Direct Analysis in Real Time (DART) Mass Spectrum of [Na(thd)] 9

1. Solid-State Structure of $[NaMn_2(thd)_4(OAc)]_2$

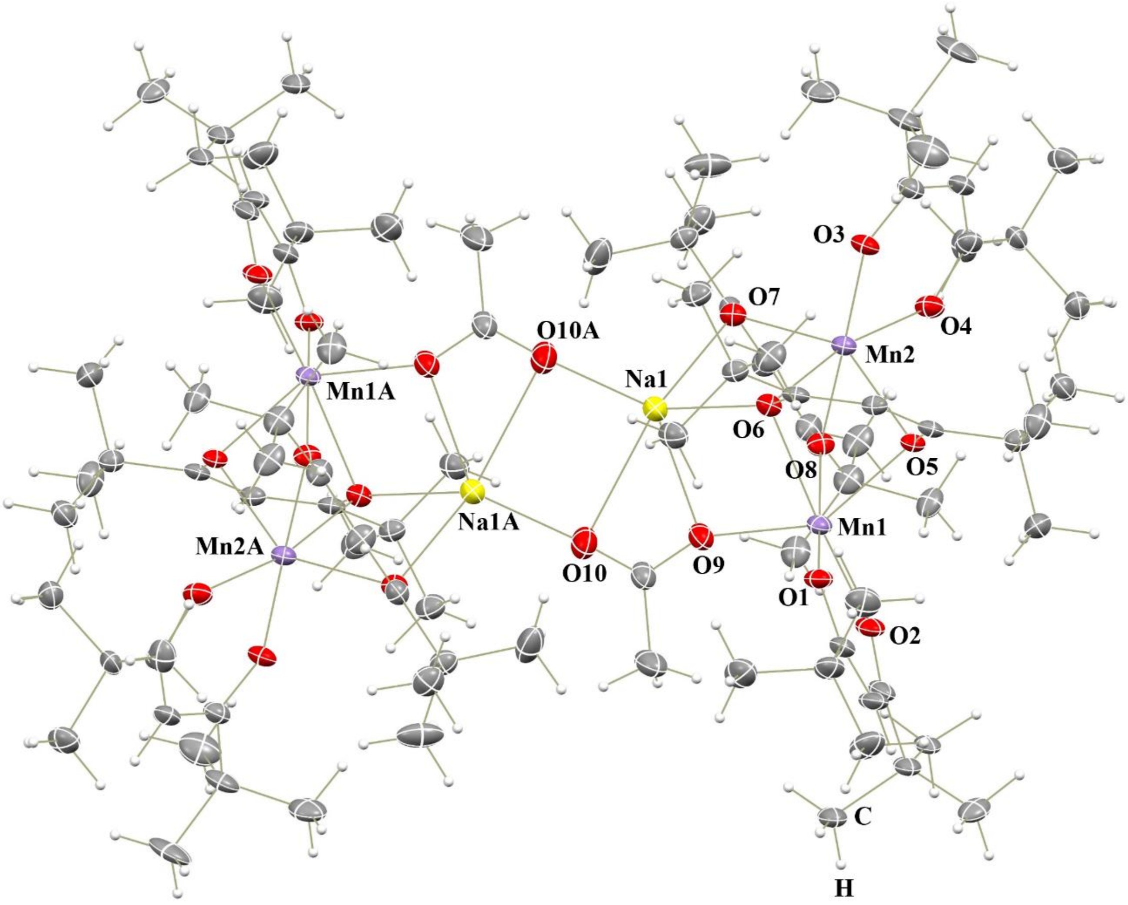

Figure S1. Molecular structure of the first crystallographically independent unit $[NaMn_2(thd)_4(OAc)]_2$ (**1**). The Na, Mn, O, and C atoms are represented by thermal ellipsoids at the 30% probability level. Hydrogen atoms are shown as spheres of arbitrary radius.

Table S1. Bond Distances (Å) and Angles (°) in the Structure of the first crystallographically independent unit $[NaMn_2(thd)_4(OAc)]_2$ (**1**).

**Bond distances**					
Na(1)-O(6)**	2.329(2)	Mn(1)-O(1)*	2.0699(19)	Mn(2)-O(3)*	2.0576(18)
Na(1)-O(7)**	2.357(2)	Mn(1)-O(2)*	2.0671(19)	Mn(2)-O(4)*	2.0637(19)
Na(1)-O(9)**	2.338(2)	Mn(1)-O(5)**	2.1807(17)	Mn(2)-O(5)**	2.2082(17)
Na(1)-O(10)**	2.635(2)	Mn(1)-O(6)**	2.5077(18)	Mn(2)-O(6)**	2.2559(18)
Na(1)-O(10A)**	2.180(2)	Mn(1)-O(8)**	2.1831(19)	Mn(2)-O(7)**	2.1324(18)
		Mn(1)-O(9)**	2.0829(19)	Mn(2)-O(8)**	2.1870(18)
**Angles**					
O(6)-Na(1)-O(7)	76.83(7)	O(1)-Mn(1)-O(5)	94.68(7)	O(3)-Mn(2)-O(4)	86.45(7)
O(6)-Na(1)-O(9)	80.54(7)	O(1)-Mn(1)-O(6)	86.28(6)	O(3)-Mn(2)-O(5)	108.66(7)
O(6)-Na(1)-O(10)	107.38(8)	O(1)-Mn(1)-O(8)	167.63(7)	O(3)-Mn(2)-O(6)	96.66(7)
O(7)-Na(1)-O(10)	160.11(8)	O(1)-Mn(1)-O(9)	98.81(8)	O(3)-Mn(2)-O(7)	99.31(7)
O(9)-Na(1)-O(7)	111.77(8)	O(2)-Mn(1)-O(1)	86.31(8)	O(3)-Mn(2)-O(8)	173.97(7)
O(9)-Na(1)-O(10)	51.66(7)	O(2)-Mn(1)-O(5)	108.41(7)	O(4)-Mn(2)-O(5)	96.47(7)
O(10A)-Na(1)-O(6)	123.88(9)	O(2)-Mn(1)-O(6)	171.23(7)	O(4)-Mn(2)-O(6)	168.35(7)
O(10A)-Na(1)-O(7)	110.53(9)	O(2)-Mn(1)-O(8)	104.84(8)	O(4)-Mn(2)-O(7)	107.56(8)
O(10A)-Na(1)-O(9)	135.04(9)	O(2)-Mn(1)-O(9)	104.19(8)	O(4)-Mn(2)-O(8)	89.52(7)
O(10A)-Na(1)-O(10)	83.70(9)	O(5)-Mn(1)-O(6)	67.56(6)	O(5)-Mn(2)-O(6)	71.90(6)
		O(5)-Mn(1)-O(8)	76.89(7)	O(7)-Mn(2)-O(5)	144.08(7)
		O(8)-Mn(1)-O(6)	82.14(6)	O(7)-Mn(2)-O(6)	83.08(7)
		O(9)-Mn(1)-O(5)	145.36(7)	O(7)-Mn(2)-O(8)	77.63(7)
		O(9)-Mn(1)-O(6)	81.60(7)	O(8)-Mn(2)-O(5)	76.24(6)
		O(9)-Mn(1)-O(8)	83.83(8)	O(8)-Mn(2)-O(6)	88.17(7)

* – chelating oxygen; ** – bridging oxygen

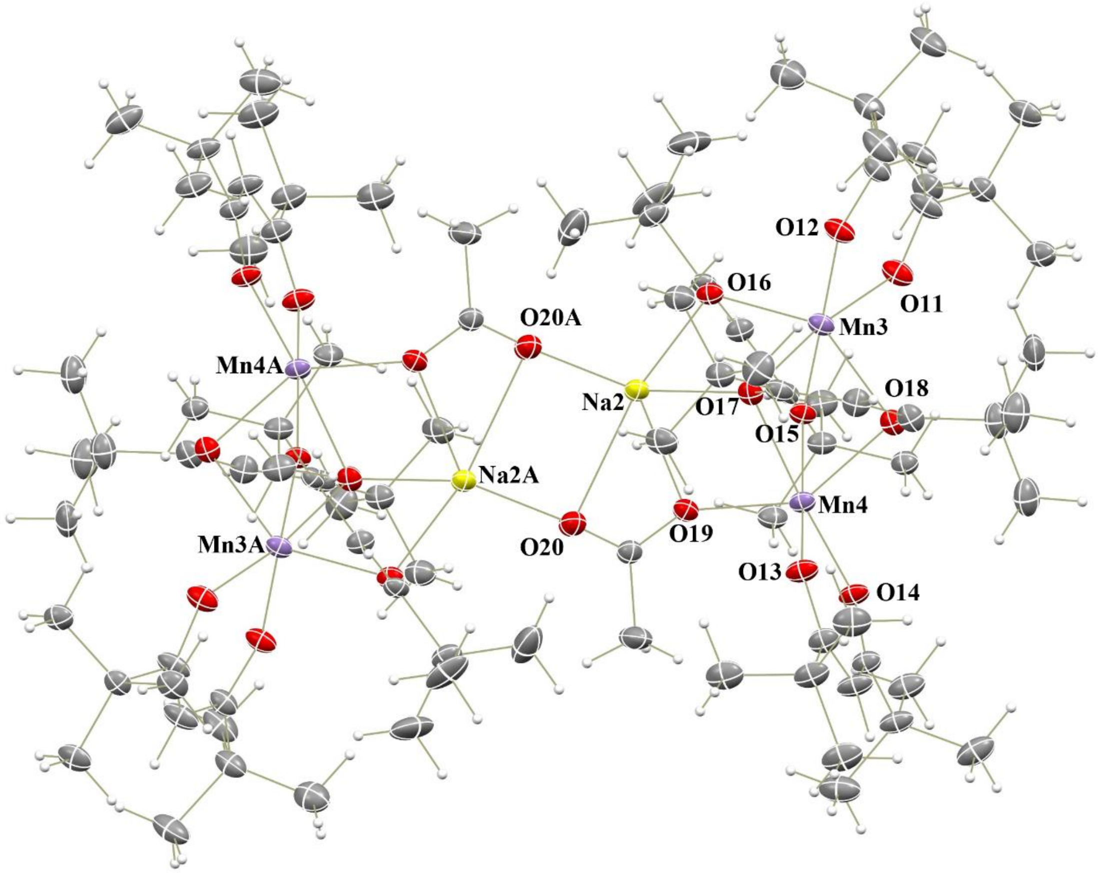

Figure S2. Molecular structure of the second crystallographically independent unit $[NaMn_2(thd)_4(OAc)]_2$ (**1**). The Na, Mn, O, and C atoms are represented by thermal ellipsoids at the 30% probability level. Hydrogen atoms are shown as spheres of arbitrary radius.

Table S2. Bond Distances (Å) and Angles (º) in the Structure of the second crystallographically independent unit $[NaMn_2(thd)_4(OAc)]_2$ (**1**).

**Bond distances**					
Na(2)-O(16)**	2.352(2)	Mn(3)-O(11)*	2.0672(18)	Mn(4)-O(13)*	2.0717(18)
Na(2)-O(17)**	2.320(2)	Mn(3)-O(12)*	2.0469(19)	Mn(4)-O(14)*	2.0676(18)
Na(2)-O(19)**	2.348(2)	Mn(3)-O(15)**	2.1708(18)	Mn(4)-O(15)**	2.1722(17)
Na(2)-O(20A)**	2.187(2)	Mn(3)-O(16)**	2.1468(19)	Mn(4)-O(17)**	2.5456(18)
Na(2)-O(20)**	2.575(2)	Mn(3)-O(17)**	2.2613(17)	Mn(4)-O(18)**	2.1914(19)
		Mn(3)-O(18)**	2.2096(19)	Mn(4)-O(19)**	2.0797(19)
**Angles**					
O(16)-Na(2)-O(20)	155.90(8)	O(11)-Mn(3)-O(15)	87.99(7)	O(13)-Mn(4)-O(15)	170.30(7)
O(17)-Na(2)-O(16)	77.87(7)	O(11)-Mn(3)-O(16)	103.63(8)	O(13)-Mn(4)-O(18)	96.91(7)
O(17)-Na(2)-O(19)	80.52(7)	O(11)-Mn(3)-O(17)	172.13(8)	O(13)-Mn(4)-O(19)	98.27(8)
O(17)-Na(2)-O(20)	107.68(7)	O(11)-Mn(3)-O(18)	100.00(8)	O(14)-Mn(4)-O(13)	86.37(7)
O(19)-Na(2)-O(16)	107.29(7)	O(12)-Mn(3)-O(11)	86.27(7)	O(14)-Mn(4)-O(15)	102.40(7)
O(19)-Na(2)-O(20)	52.58(7)	O(12)-Mn(3)-O(15)	172.79(7)	O(14)-Mn(4)-O(18)	108.74(8)
O(20A)-Na(2)-O(16)	113.18(8)	O(12)-Mn(3)-O(16)	99.16(8)	O(14)-Mn(4)-O(19)	105.06(8)
O(20A)-Na(2)-O(17)	122.61(9)	O(12)-Mn(3)-O(17)	96.01(7)	O(15)-Mn(4)-O(18)	76.49(7)
O(20A)-Na(2)-O(19)	136.47(8)	O(12)-Mn(3)-O(18)	109.17(8)	O(17)-Mn(4)-O(13)	87.79(7)
O(20A)-Na(2)-O(20)	84.13(8)	O(15)-Mn(3)-O(17)	90.26(7)	O(17)-Mn(4)-O(14)	172.23(8)
		O(15)-Mn(3)-O(18)	76.14(7)	O(17)-Mn(4)-O(15)	83.10(7)
		O(16)-Mn(3)-O(15)	77.96(7)	O(17)-Mn(4)-O(18)	67.04(7)
		O(16)-Mn(3)-O(17)	83.49(7)	O(17)-Mn(4)-O(19)	80.72(7)
		O(16)-Mn(3)-O(18)	144.01(7)	O(19)-Mn(4)-O(15)	83.57(7)
		O(18)-Mn(3)-O(17)	72.14(7)	O(19)-Mn(4)-O(18)	143.62(7)

* – chelating oxygen; ** – bridging oxygen

2. IR Spectrum of $[NaMn_2(thd)_4(OAc)]_2$

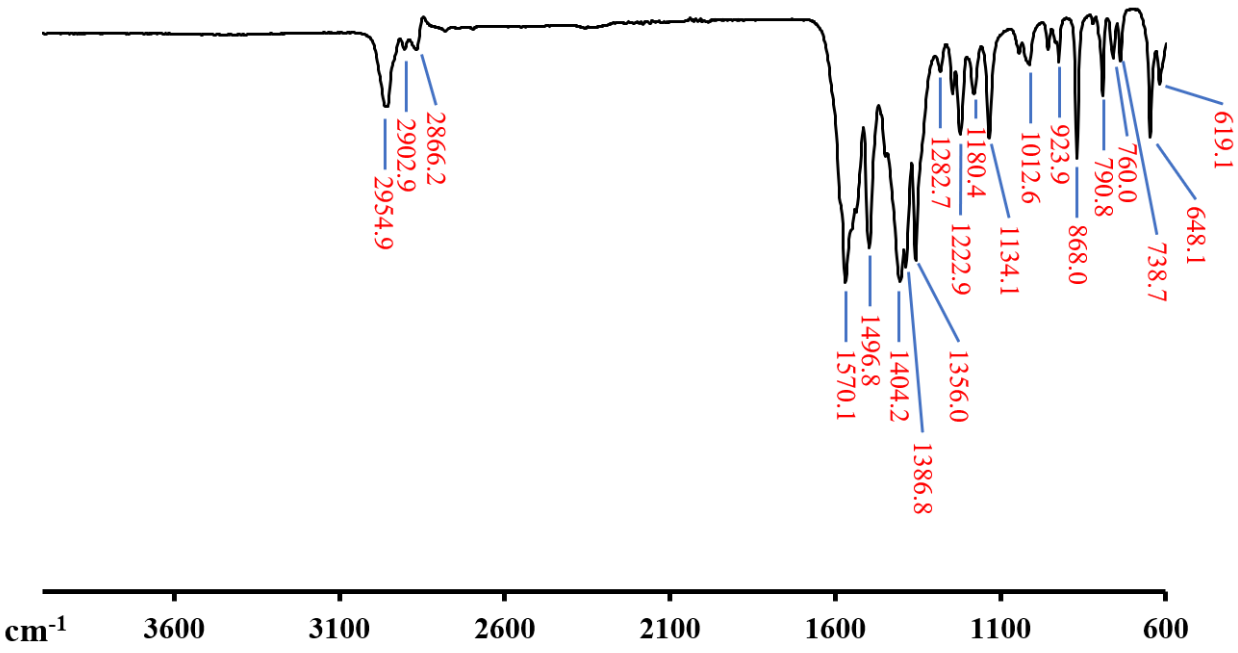

Figure S3. IR spectrum of $[NaMn_2(thd)_4(OAc)]_2$ (**1**).

3. ^{1}H NMR Spectrum of [Na(thd)]

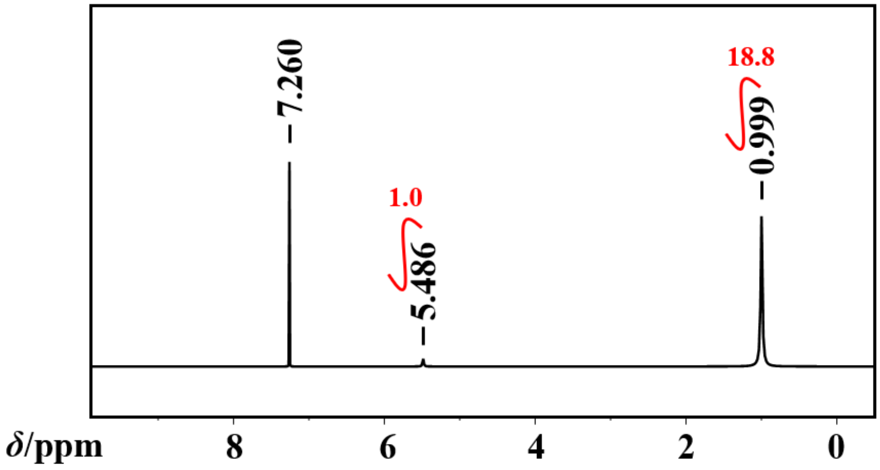

Figure S4. ^{1}H NMR spectrum of [Na(thd)] in $CDCl_3$.

4. Direct Analysis in Real Time (DART) Mass Spectrum of [Na(thd)]

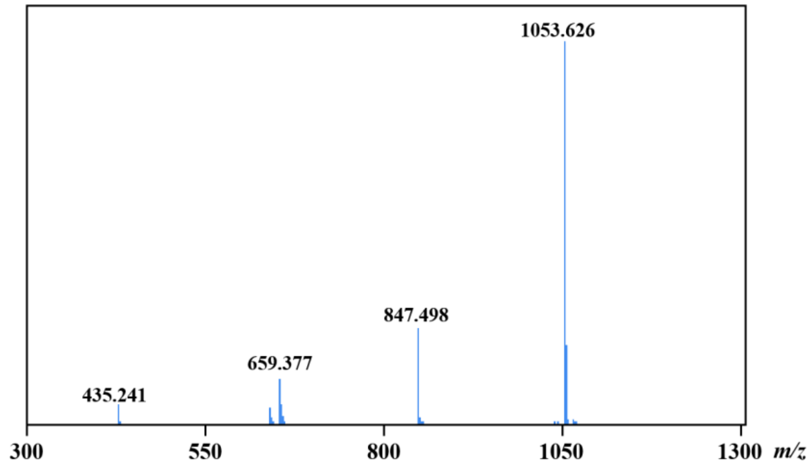

Figure S5. Positive-ion DART mass spectrum of [Na(thd)] measured at 350 ºC.

Table S3. Assignment of ions detected in a positive-ion DART mass spectrum of [Na(thd)]. All ions having relative intensity higher than 3% are shown in the table.

**Fragments**	**Calc. *m/z***	**Exp. *m/z***	***Δ***	**Relative Int. (%)**
$[Na_6(thd)_5]^+$	1053.631	1053.626	0.005	100
$[Na_5(thd)_4]^+$	847.503	847.498	0.005	24.9
$[Na_4(thd)_3(H_2O)]^+$	659.385	659.377	0.008	15.7
$[Na_4(thd)_3]^+$	641.375	641.370	0.005	4.3
$[Na_3(thd)_2]^+$	435.246	435.241	0.005	5.1
